# MicroRNA-203 negatively regulates c-Abl, ERK1/2 phosphorylation, and proliferation in smooth muscle cells

**DOI:** 10.14814/phy2.12541

**Published:** 2015-09-23

**Authors:** Guoning Liao, Reynold A Panettieri, Dale D Tang

**Affiliations:** 1 The Center for Cardiovascular Sciences, Albany Medical CollegeAlbany, New York; 2 Department of Medicine, University of PennsylvaniaPhiladelphia, Pennsylvania

**Keywords:** Cell proliferation, ERK1/2, microRNA, smooth muscle, tyrosine kinase

## Abstract

The nonreceptor tyrosine kinase c-Abl has a role in regulating smooth muscle cell proliferation, which contributes to the development of airway remodeling in chronic asthma. MicroRNAs (miRs) are small noncoding RNA molecules that regulate gene expression by binding to complementary sequences in the 3′ untranslated regions (3′ UTR) of target mRNAs. Previous analysis suggests that miR-203 is able to bind to the 3′ UTR of human c-Abl mRNA. In this report, treatment with miR-203 attenuated the expression of c-Abl mRNA and protein in human airway smooth muscle (HASM) cells. Furthermore, transfection with an miR-203 inhibitor enhanced the expression of c-Abl at mRNA and protein levels in HASM cells. Treatment with platelet-derived growth factor (PDGF) induced the proliferation and ERK1/2 phosphorylation in HASM cells. Exposure to miR-203 attenuated the PDGF-stimulated proliferation and ERK1/2 phosphorylation in HASM cells. The expression of c-Abl at protein and mRNA levels was higher in asthmatic HASM cells, whereas the level of miR-203 was reduced in asthmatic HASM cells as compared to control HASM cells. Taken together, our present results suggest that miR-203 is a negative regulator of c-Abl expression in smooth muscle cells. miR-203 regulates smooth muscle cell proliferation by controlling c-Abl expression, which in turn modulates the activation of ERK1/2.

## Introduction

c-Abl (Abelson tyrosine kinase, Abl, ABL1) is a nonreceptor tyrosine kinase that has a role in the regulation of the actin cytoskeleton important for various cellular functions including cell adhesion and migration as well as cardiac growth and development (Hu et al. 2005; Qiu et al. 2010; Ring et al. 2011; Cleary et al. 2014). In smooth muscle, c-Abl is necessary for force development in response to contractile activation (Anfinogenova et al. 2007; Chen et al. 2009; Jia and Tang 2010; Cleary et al. 2013). More importantly, c-Abl gets phosphorylated and activated in smooth muscle cells in response to stimulation with growth factors. Activated c-Abl promotes actin polymerization, which regulates the recruitment and activation of Raf-1. Activated Raf-1 subsequently promotes phosphorylation of MEK1/2 and ERK1/2, which eventually enhances smooth muscle cell proliferation (Widmann et al. 1999; Vallieres et al. 2009; Jia et al. 2012; Wang et al. 2013a). Interestingly, c-Abl does not affect the activation of AKT, another protein kinase implicated in cell growth and survival (Orsini et al. 1999; Jia et al. 2012; Wang et al. 2013a).

Animal studies suggest that c-Abl may contribute to the pathogenesis of asthma (Cleary et al. 2013; Wang et al. 2014a). c-Abl expression is upregulated in airway smooth muscle tissues from an animal model of asthma as well as in asthmatic airway smooth muscle cells. Conditional knockout of c-Abl inhibits the allergen-induced airway remodeling in an animal model of asthma (Cleary et al. 2013). Moreover, treatment with the c-Abl inhibitor imatinib also attenuated airway thickening in a chronic animal model of asthma (Rhee et al. 2011). The mechanisms that control c-Abl expression in smooth muscle cells are largely unknown.

MicroRNAs (miRNAs) are evolutionarily conserved, 18–25 nucleotides, noncoding RNA molecules that belong to a novel class of gene regulators and control gene expression by binding to complementary sequences in the 3′ untranslated regions (3′ UTR) of target mRNAs, which may lead to target mRNA degradation and/or translational repression (Bartel 2004; Joshi et al. 2011). miRNAs are first transcribed from the genome as primary transcripts (pri-miRNAs), which are cleaved to hairpin-structured precursors (pre-miRNAs) by the complex composed of Drosha, DGCR8, and cofactors. Pre-miRNAs are then translocated into the cytoplasm and processed by Dicer to become mature miRNAs (Bartel 2004; Jude et al. 2012). miRNAs have been implicated in cell proliferation, differentiation (Albinsson et al. 2010), tumorigenesis (Bueno et al. 2008; Craig et al. 2011), and calcium-related proteins (Jude et al. 2012).

miR-203 has been implicated in the pathogenesis of T-cell lymphomas. The expression of miR-203 was reduced in murine T-cell lymphomas. However, the expression of c-Abl was higher in these tumor cells (Bueno et al. 2008). The introduction of miR-203 inhibited the expression of c-Abl and BCR-c-Abl in tumor cells, and attenuated tumor cell proliferation in c-Abl-dependent manner. Using an c-Abl mRNA 3′ UTR reporter, Bueno et al. demonstrated that miR-203 directly binds to the 3′ UTR of c-Abl mRNA (Bueno et al. 2008; Craig et al. 2011).

The objective of this study was to evaluate whether miR-203 is involved in the regulation of c-Abl expression in smooth muscle cells. Furthermore, we examined the role of miR-203 in ERK1/2 phosphorylation and the proliferation in smooth muscle cells in response to activation with a growth factor.

## Materials and Methods

### Cell culture

Human airway smooth muscle (HASM) cells were prepared from bronchi and adjacent tracheas of control subjects (died from nonasthmatic causes) and patients (died from severe asthma) obtained from the International Institute for Advanced Medicine (Wang et al. 2013a). Asthmatic HASM cells were also obtained from Dr. Reynold A. Panettieri of University of Pennsylvania (Jude et al. 2012). Human tissues were nontransplantable and consented for research. This study was approved by the Albany Medical College Committee on research involving human subjects. Briefly, muscle tissues were incubated for 20 min with dissociation solution (130 mmol/L NaCl, 5 mmol/L KCl, 1.0 mmol/L CaCl_2_, 1.0 mmol/L MgCl_2_, 10 mmol/L Hepes, 0.25 mmol/L EDTA, 10 mmol/L d-glucose, 10 mmol/L taurine, pH 7, 4.5 mg collagenase [type I], 10 mg papain [type IV], 1 mg/mL BSA, and 1 mmol/L dithiothreitol). All enzymes were purchased from Sigma-Aldrich (St. Louis, MO). The tissues were then washed with Hepes-buffered saline solution (composition in mmol/L: 10 Hepes, 130 NaCl, 5 KCl, 10 glucose, 1 CaCl_2_, 1 MgCl_2_, 0.25 EDTA, 10 taurine, pH 7). The cell suspension was mixed with Ham’s F12 medium supplemented with 10% (v/v) fetal bovine serum (FBS) and antibiotics (100 units/mL penicillin, 100 *μ*g/mL streptomycin). Cells were cultured at 37°C in the presence of 5% CO_2_ in the same medium. The medium was changed every 3–4 days until cells reached confluence, and confluent cells were passaged with trypsin/EDTA solution (Li et al. 2006, 2009; Wang et al. 2013a; Cleary et al. 2014). Passage 5–10 of smooth muscle cells were used for the studies. Cell lines from five control subjects and four asthmatic subjects were available for the experiments. In some cases, duplicated experiments were performed for a cell line from a donor.

### Assessment of mRNA expression

Total RNA was isolated by using the High Pure RNA Isolation Kit (Roche, Indianapolis, IN). The levels of mRNA were determined by reverse transcription quantitative real-time polymerase chain reaction (RT-qPCR). For the detection of human c-Abl mRNA, the 5′-primer sequence was 5′-AGCTCTACGTCTCCTCCGAG-3′ and the 3′-primer sequence was 5′-CAGCTTGTGCTTCATGGTGA-3′. Human *β*2-microglobulin (B2M) mRNA was used as a control. The 5′-primer sequence of B2M was 5′-TGCTGTCTCCATGTTTGATGTATCT-3′ and the 3′-primer sequence of B2M was 5′-TCTCTGCTCCCCACCTCTAAGT-3′. Briefly, total RNA and primers were mixed with the iTaq Universal SYBR Green One-Step Kit (Bio-Rad, Hercules, CA) and the mRNA levels were detected using a real-time PCR detection system (Bio-Rad). The expression level of c-Abl mRNA was expressed as the ratio of c-Abl mRNA over B2M mRNA.

### Measurement of miRNA expression

The NCode miRNA First-Strand cDNA Synthesis Kit (Life Technologies, Carlsbad, CA) was used to generate poly A tailing of miRNAs from purified total RNA and the first-strand cDNA. Afterward, the real-time PCR (qPCR) amplification was performed by using the SsoAdvanced Universal SYBR Green Supermix kit (Bio-Rad). Human miR-203 (MIMAT0000264) 5′-primer, U6-2 housekeeping gene 5′-primer, and the universal 3′ miRNA primer were purchased from Applied Biological Materials, Inc. (Richmond, BC, Canada). The expression level of miR-203 was expressed as the ratio of miR-203 over U6-2 RNA.

### Cell transfection

miR-203 (CAT#4464066), miR-control (CAT#4464058), miR-203 inhibitor (CAT#4464084), and miR inhibitor negative control (CAT#4464076) were purchased from Ambion/Life Technologies. Cell transfection was performed by using the lipofectamine 2000 reagent (Invitrogen/Life Technologies, Carlsbad, CA) according to the manufacture’s manual.

### Assessment of cell proliferation

Cell (4.8 × 10^4^) were plated in the F12 medium supplemented with 10% FBS (Invitrogen) for ≥18 h. Cells were then transfected with miR-203 mimic or miR-control. They are subsequently treated with human platelet-derived growth factor (PDGF)-BB (Sigma, 10 ng/mL) in the F12 medium containing 0.25% FBS. Additional cells were cultured in the medium with 0.25% FBS as a control. Numbers of viable cells were evaluated using the trypan blue exclusion test. Triplicate samples were averaged for each experiment.

### Immunoblot analysis

Immunoblot analysis was performed using the experimental procedures as previously described (Wang et al. 2013b, 2014a,b; Chen and Tang 2014; Cleary et al. 2014; Li et al. 2014). Antibodies against c-Abl and total ERK1/2, phospho-AKT (Ser-473), and total AKT were purchased from Cell Signaling Technology (Danvers, MA). Phospho-ERK1/2 (Thr202/Tyr204) antibody was purchased from Santa Cruz Biotechnology (Dallas, TX) and glyceraldehyde-3-phosphate dehydrogenase (GAPDH) antibody was purchased from Ambion/ThermoFisher Scientific (Grand Island, NY). The levels of phosphoprotein and total protein were quantified by scanning densitometry of immunoblots (Fuji Multigauge Software, Valhalla, NY).

### Statistical analysis

All statistical analysis was performed using Prism 6 software (GraphPad Software, San Diego, CA). Differences between two groups were analyzed by Student–Newman–Keuls test or Dunn’s method. Values of *n* refer to the number of experiments used to obtain each value. *P* < 0.05 was considered to be statistical significant.

## Result

### miR-203 downregulates the expression of c-Abl in HASM cells

**Figure 1:**
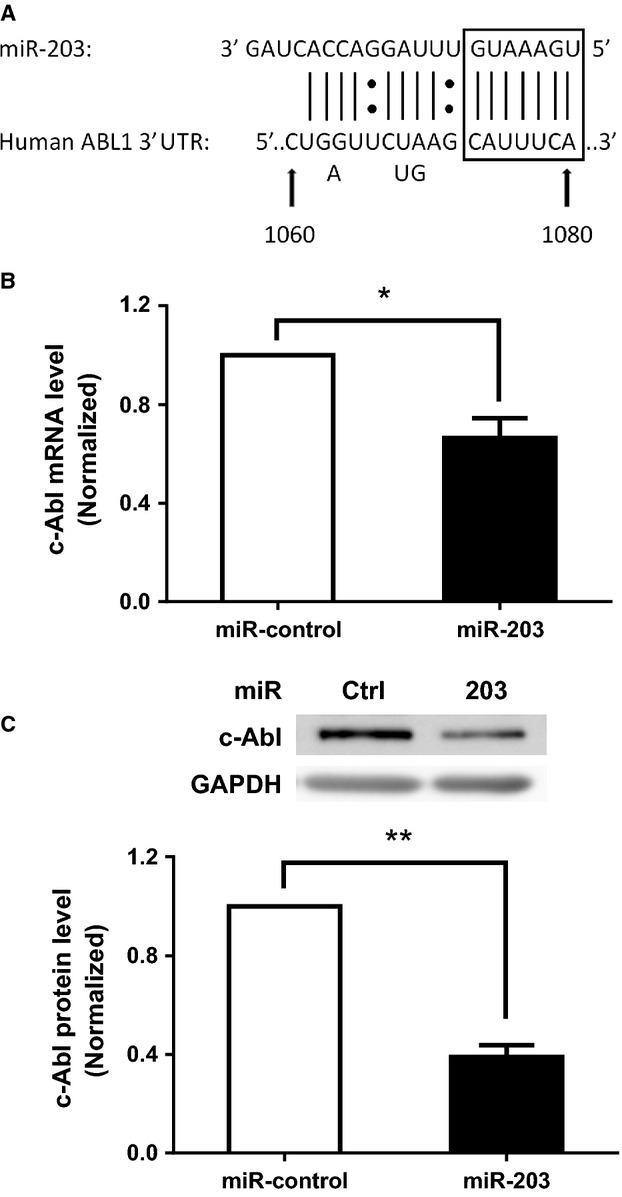
Treatment with miR-203 inhibits the expression of c-Abl at mRNA and protein levels in human airway smooth muscle (HASM) cells. (A) Sequence alignment between miR-203 and 3′ untranslated region (UTR) of human c-Abl mRNA. The seed region is in the box. (B) mRNA of c-Abl in HASM cells transfected with either 20 nmol/L miR-control or miR-203 for 3 days was assessed by RT-qPCR (reverse transcription quantitative real-time polymerase chain reaction). (C) Blots of HASM cells transfected with either miR-control or miR-203 for 3 days were probed with antibodies against c-Abl and glyceraldehyde-3-phosphate dehydrogenase (GAPDH). Values represent mean ± SD (*P < 0.05, **P < 0.01, *n* = 4).

miRNA sequence analysis suggests that miR-203 is likely to target the 3′ UTR of c-Abl (Bueno et al. 2008) (Fig. 1A). To assess the role of miR-203 in smooth muscle cells, HASM cells were transfected with miR-203 mimics or miR-control for 3 days. miRNA mimics are small, chemically modified double-stranded RNA molecules that are designed to mimic endogenous mature miRNAs. The expression of c-Abl mRNA and protein in these cells was evaluated by RT-qPCR and western blotting, respectively. Compared to miR-control, transfection with miR-203 attenuated the expression of c-Abl in HASM cells at mRNA (Fig. 1B) and protein (Fig. 1C) levels. The results suggest that miR-203 is able to degrade c-Abl mRNA and protein.

### Treatment with miR-203 inhibitor increases the expression of c-Abl in HASM cells

**Figure 2:**
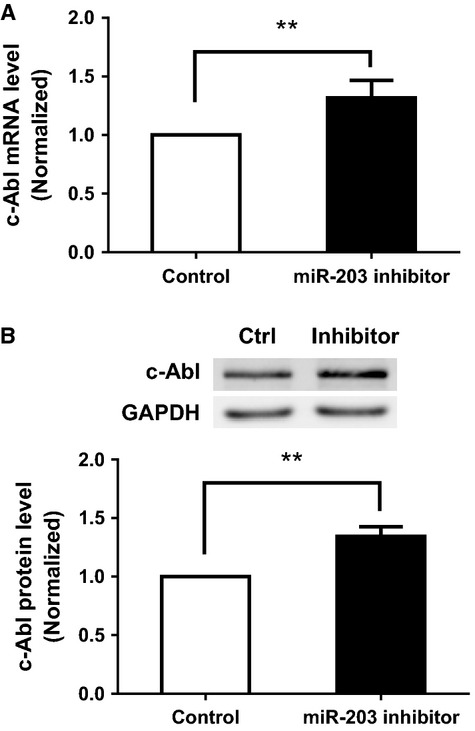
Treatment with miR-203 inhibitor increases the expression of c-Abl in human airway smooth muscle (HASM) cells. (A) HASM cells were transfected with either 20 nmol/L control RNA or miR-203 inhibitor (see Materials and Methods section). miR-203 inhibitor is small, chemically modified single-stranded RNA molecules designed to specifically bind to and inhibit endogenous miR-203. mRNA of c-Abl in these cells was assessed by RT-qPCR (reverse transcription quantitative real-time polymerase chain reaction) 2 days after transfection. (B) Blots of HASM cells transfected with either 20 nmol/L control RNA or miR-203 inhibitor for 2 days were probed with antibodies against c-Abl and glyceraldehyde-3-phosphate dehydrogenase (GAPDH). Values represent mean ± SD (**P < 0.01, *n* = 5).

To assess whether endogenous miR-203 has a role in regulating c-Abl, HASM cells were transfected with either 20 nmol/L miR-203 inhibitor or negative control for miR inhibitor. miRNA inhibitors are small, chemically modified single-stranded RNA molecules designed to specifically bind to and inhibit endogenous miRNA molecules. Two days after transfection, mRNA and protein levels of c-Abl in these cells were then assessed. The introduction of miR-203 inhibitor resulted in an increase in c-Abl mRNA and protein in these cells (Fig. 2).

### Treatment with miR-203 inhibits the PDGF-induced proliferation of HASM cells

**Figure 3:**
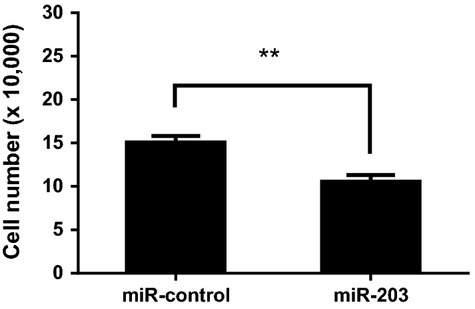
Treatment with miR-203 attenuates the platelet-derived growth factor (PDGF)-induced proliferation of human airway smooth muscle (HASM) cells. HASM cells were transfected with either miR-control or miR-203. One day after transfection, they were treated with 10 ng/mL PDGF for 3 days. The numbers of viable cells were then determined. Exposure to miR-203 inhibits the PDGF-induced proliferation in HASM cells. Data are mean ± SD (**P < 0.01, *n* = 5).

Since miR-203 is able to regulate the expression of c-Abl (Figs. 1, 2), and c-Abl has been implicated in smooth muscle cell proliferation (Jia et al. 2012; Wang et al. 2013a; Chen and Tang 2014), we questioned whether miR-203 affects smooth muscle cell proliferation. HASM cells were transfected with either miR-control or miR-203. One day after transfection, cells were stimulated with 10 ng/mL PDGF, or left unstimulated for 3 days. Treatment with miR-203 attenuated the PDGF-induced cell proliferation as compared to cells treated with miR-control (Fig. 3).

### PDGF-induced ERK1/2 phosphorylation is reduced in HASM cells treated with miR-203

**Figure 4:**
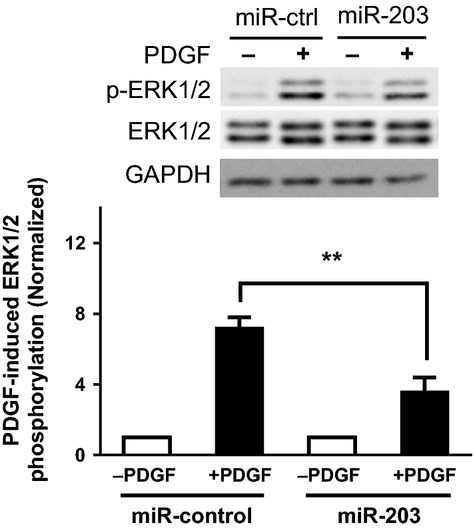
Exposure to miR-203 inhibits the platelet-derived growth factor (PDGF)-induced phosphorylation of ERK1/2. Representative western blots illustrating the effects of miR-203 on ERK1/2 phosphorylation (Thr202/Tyr204) in human airway smooth muscle (HASM) cells. Cells transfected with miR-control or miR-203 were stimulated with 10 ng/mL PDGF for 10 min or they were not stimulated. ERK1/2 phosphorylation in these cells was evaluated by immunoblot analysis. Treatment with miR-203 attenuates the PDGF-induced ERK1/2 phosphorylation. The phosphorylation levels of ERK1/2 in cells stimulated with PDGF are normalized to corresponding unstimulated levels. Data are mean ± SD (**P < 0.01, *n* = 4). miR-ctrl, miR-control.

As described earlier, ERK1/2 phosphorylation plays a critical role in the signaling pathways that control cell proliferation (Jia et al. 2012; Wang et al. 2013a). It has been shown that PDGF treatment for 10 min significantly induced ERK1/2 phosphorylation in smooth muscle cells (Orsini et al. 1999; Jia et al. 2012; Wang et al. 2013a). We assessed the effects of miR-203 on ERK1/2 phosphorylation in HASM cells. Cells transfected with miR-control or miR-203 were stimulated with 10 ng/mL PDGF for 10 min or they were not stimulated. ERK1/2 phosphorylation in these cells was evaluated by immunoblot analysis. In cells treated with miR-control, PDGF stimulation induced ERK1/2 phosphorylation. However, the PDGF-induced ERK1/2 phosphorylation was reduced in HASM cells treated with miR-203 (Fig. 4). Exposure to miR-203 did not attenuate ERK1/2 phosphorylation in cells without PDGF stimulation. Moreover, the expression of total ERK1/2 in cells was not affected by treatment with miR-203.

### Treatment with miR-203 does not affect AKT phosphorylation

**Figure 5:**
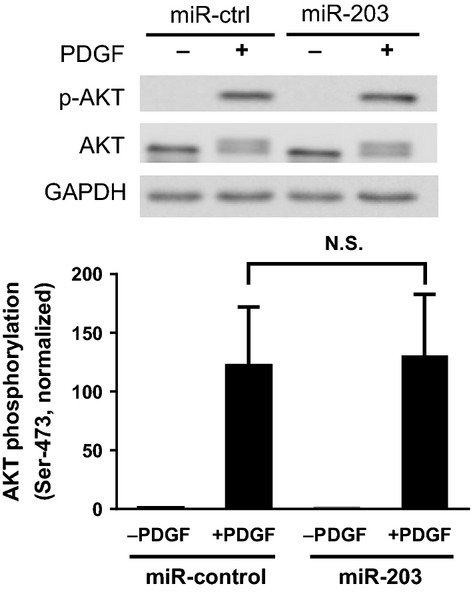
Treatment with miR-203 does not affect AKT phosphorylation in cells. Representative western blots illustrating the effects of miR-203 on AKT phosphorylation (Ser-473). Human airway smooth muscle (HASM) cells transfected with miR-control or miR-203 were stimulated with 10 ng/mL platelet-derived growth factor (PDGF) for 10 min or they were not stimulated followed by immunoblot analysis. Treatment with miR-203 does not influence the PDGF-induced AKT phosphorylation. Please notice that phosphorylated AKT displays a band shift on immunoblots. The phosphorylation levels of AKT in cells stimulated with PDGF are normalized to corresponding unstimulated levels. Data are mean ± SD (*P* > 0.05, *n* = 4). miR-ctrl, miR-control. N.S., not significant.

In addition to ERK1/2 activation, exposure to growth factors also activates AKT/PKB in smooth muscle cells (Hers et al. 2011; Jia et al. 2012). To determine whether AKT activation is regulated by miR-203, we assessed the effects of miR-203 on AKT phosphorylation in cells. Stimulation with PDGF induced a significant increase in AKT phosphorylation in cells treated with miR-control or miR-203 (Fig. 5). The average increase in AKT phosphorylation in these cells was not significantly different 10 min after PDGF stimulation (Fig. 5). The results suggest that miR-203 does not affect AKT activation in smooth muscle cells. Furthermore, the expression of total AKT in cells was not affected by treatment with miR-203.

### The expression of miR-203 is reduced in asthmatic HASM cells

**Figure 6:**
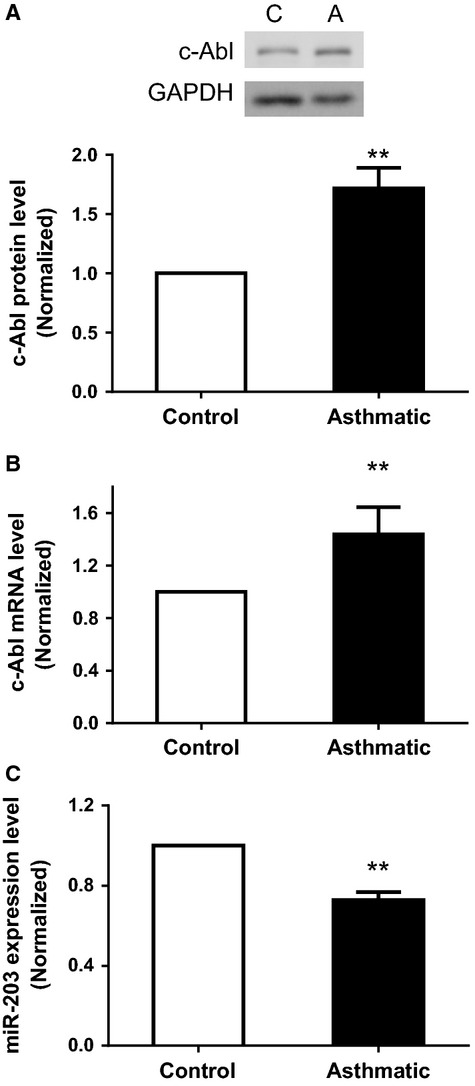
The expression of c-Abl protein, mRNA, and miR-203 in control and asthmatic human airway smooth muscle (HASM) cells. (A) Extracts of HASM cells from control subjects and patients with severe asthma were blotted with antibodies against c-Abl and glyceraldehyde-3-phosphate dehydrogenase (GAPDH). The protein levels of c-Abl in asthmatic HASM cells are higher than those in control HASM cells. C, control cells; A, asthmatic cells. (B) The mRNA levels of c-Abl from control and asthmatic HASM cells were determined by reverse transcription quantitative PCR (RT-qPCR). The mRNA levels of c-Abl in asthmatic HASM cells are higher compared to control HASM cells. (C) miR-203 levels of control and asthmatic HASM cells were evaluated by qPCR using specific primers. miR-203 level is reduced in asthmatic HASM cells. All data are mean ± SD (**P < 0.01). Control cells (*n* = 5), asthmatic cells (*n* = 5).

Because c-Abl has been implicated in the pathogenesis of asthma (Cleary et al. 2013), we evaluated the protein level of c-Abl in control and asthmatic HASM cells by immunoblot analysis. The amount of c-Abl was higher in asthmatic airway smooth muscle cells than in control airway smooth muscle cells. The ratios of c-Abl/GAPDH in asthmatic HASM cells were increased as compared to control HASM cells (Fig. 6A). The result is consistent with our previous findings in asthmatic airway smooth muscle cells/tissues (Cleary et al. 2013). We also determined the levels of c-Abl mRNA in control and asthmatic HASM cells by RT-qPCR. The level of c-Abl mRNA was higher in asthmatic HASM cells than in control HASM cells (Fig. 6B). The results suggest that both c-Abl mRNA and protein are upregulated in asthmatic HASM cells.

As shown in Figures 1 and 2, miR-203 is able to regulate c-Abl in smooth muscle cells. We compared the expression of miR-203 in control and asthmatic HASM cells by the miR-specific qPCR assay. The U6-2 housekeeping gene was used as a loading control. The expression level of miR-203 was expressed as the ratio of miR-203 over U6-2 RNA. The level of miR-203 in asthmatic HASM cells was normalized to control HASM cells. The expression of miR-203 was lower in asthmatic HASM cells as compared to control HASM cells (Fig. 6C).

### Treatment with miR-203 inhibits proliferation and ERK1/2 phosphorylation in asthmatic smooth muscle cells

**Figure 7:**
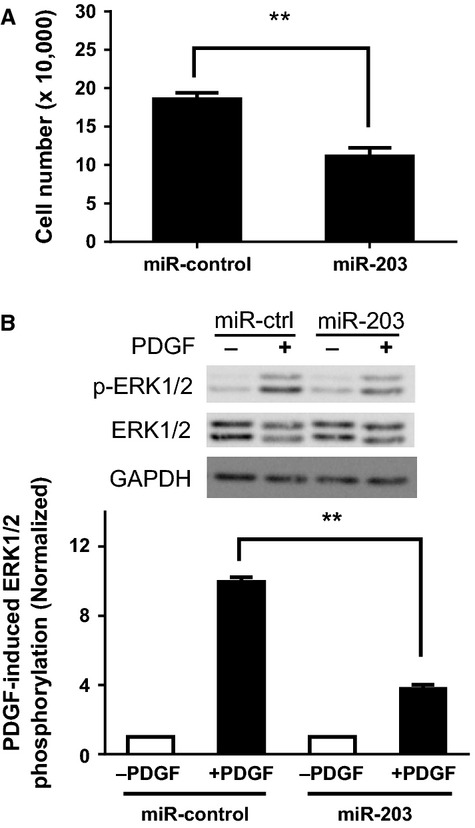
Treatment with miR-203 attenuates the platelet-derived growth factor (PDGF)-induced proliferation and ERK1/2 phosphorylation in asthmatic human airway smooth muscle (HASM) cells. (A) Asthmatic HASM cells transfected with either miR-control or miR-203 were stimulated with 10 ng/mL PDGF for 3 days. The numbers of viable cells were then determined. Exposure to miR-203 inhibits the PDGF-induced proliferation in asthmatic HASM cells (*n* = 5). Data are mean ± SD (**P < 0.01). (B) Representative western blots illustrating the effects of miR-203 on ERK1/2 phosphorylation in asthmatic HASM cells. Cells transfected with miR-control or miR-203 were stimulated with 10 ng/mL PDGF for 10 min or they were not stimulated. ERK1/2 phosphorylation in these cells was evaluated by immunoblot analysis. Treatment with miR-203 attenuates the PDGF-induced ERK1/2 phosphorylation in asthmatic cells. The phosphorylation levels of ERK1/2 in cells stimulated with PDGF are normalized to corresponding unstimulated levels. Data are mean ± SD (**P < 0.01, *n* = 4).

We also determined the effects of miR-203 on asthmatic HASM cell proliferation. The PDGF-induced proliferation was attenuated in asthmatic HASM cells transfected with miR-203 (Fig. 7A). Moreover, the effects of miR-203 on ERK1/2 phosphorylation in smooth muscle cells were evaluated. PDGF stimulation increased ERK1/2 phosphorylation in asthmatic HASM cells treated with miR-control. In contrast, treatment with miR-203 inhibited the PDGF-induced ERK1/2 phosphorylation in asthmatic HASM cells (Fig. 7B). Exposure to miR-203 did not blunt basal ERK1/2 phosphorylation in asthmatic cells.

## Discussion

The nonreceptor tyrosine kinase c-Abl has a physiological role in regulating cell proliferation, cytokinesis, migration, and smooth muscle contraction (Anfinogenova et al. 2007; Wang et al. 2013a,b, 2014a; Chen and Tang 2014; Cleary et al. 2014). In addition, c-Abl has been implicated in the pathogenesis of airway remodeling, a key feature of asthma (Rhee et al. 2011; Cleary et al. 2013). Furthermore, c-Abl expression is altered in an animal model of asthma and in asthmatic airway smooth muscle cells (Cleary et al. 2013). The mechanisms that control c-Abl expression in smooth muscle cells have been elusive.

miRNAs are small noncoding RNA molecules that regulate gene expression by binding to complementary sequences in the 3′ UTR of target mRNAs (Bartel 2004). miR sequence analysis suggests that miR-203 is able to bind to the 3′ UTR of human c-Abl mRNA, which is verified by studies on a c-Abl mRNA 3′ UTR reporter (Bueno et al. 2008). In this report, treatment with miR-203 attenuated the expression of c-Abl in smooth muscle cells. Moreover, exposure to the miR-203 inhibitor, which is able to bind to and block endogenous miR-203, increased the level of c-Abl in these cells. These results suggest that miR-203 is able to negatively regulate the expression of c-Abl in smooth muscle cells.

The complementary binding of miRNAs to the 3′ UTR of target mRNA may lead to target mRNA degradation and/or translational repression (Bartel 2004). In the present study, treatment with miR-203 reduced the expression of c-Abl mRNA by 35%. However, exposure to miR-203 attenuated the level of c-Abl protein by 60%. These results suggest that miR-203 regulates c-Abl expression by affecting both mRNA degradation and protein translation. miR-203 may bind to complementary sequences in the 3′ UTR of c-Abl, which activates the RNA-induced silencing complex and induces c-Abl mRNA degradation (Bartel 2004). In addition, the binding of miR-203 to the 3′ UTR of c-Abl may repress the translation of c-Abl in cells (Bartel 2004; Joshi et al. 2011). Furthermore, treatment with miR-203 mimic did not affect the expression of ERK1/2 and AKT in cells, suggesting the selectivity of miR-203 mimic.

In this study, treatment with PDGF induced smooth muscle cell proliferation, which is consistent with our previous studies (Jia et al. 2012; Wang et al. 2013a). Moreover, the introduction of miR-203 inhibited the PDGF induced smooth muscle cell proliferation. To the best of our knowledge, this is the first evidence that miR-203 is capable of regulating smooth muscle cell proliferation.

ERK1/2 phosphorylation plays a critical role in the cellular processes that control cell proliferation (Widmann et al. 1999; Jia et al. 2012; Wang et al. 2013a). Our previous studies demonstrate that c-Abl regulates smooth muscle cell proliferation by controlling ERK1/2 activation (Jia et al. 2012; Wang et al. 2013a). In this report, the treatment with miR-203 inhibited the PDGF-induced ERK1/2 phosphorylation in smooth muscle cells. However, exposure to miR-203 did not affect AKT phosphorylation in smooth muscle cells. AKT phosphorylation has been implicated in cell growth and survival (Orsini et al. 1999; Jia et al. 2012; Wang et al. 2013a). Since miR-203 is able to diminish the expression of c-Abl, ERK1/2 phosphorylation, and cell proliferation, it is likely that miR-203 regulates c-Abl expression, which in turn affects ERK1/2 activation and smooth muscle cell proliferation. However, we do not rule out the possibility that miR-203 may also affect other cellular processes (such as programmed cell death), which may also reduce the PDGF-induced increase in cell numbers. Future studies are needed to test the possibility.

In the present study, the expression of c-Abl at protein and mRNA levels is increased in asthmatic airway smooth muscle cells, which is consistent with our previous findings (Cleary et al. 2013). Furthermore, the levels of miR-203 were diminished in asthmatic airway smooth muscle cells. Because c-Abl expression is upregulated in these cells, this raises the possibility that reduced miR-203 expression may be associated with the upregulation of c-Abl in asthmatic airway smooth muscle cells. Moreover, the introduction of miR-203 attenuated the growth factor-induced ERK1/2 phosphorylation and the proliferation in asthmatic HASM cells. The results suggest that miR-203 is also able to blunt the growth factor-mediated pathway and proliferation in asthmatic airway smooth muscle cells.

In summary, our present results suggest that miR-203 is a negative regulator of c-Abl expression in smooth muscle cells. miR-203 regulates smooth muscle cell proliferation by controlling c-Abl expression, which subsequently modulates the activation of ERK1/2.
